# Comparison of Metacognitions in Obsessive-Compulsive Disorder, Generalized Anxiety Disorder, and Healthy Controls

**DOI:** 10.1192/j.eurpsy.2022.488

**Published:** 2022-09-01

**Authors:** I. Gundogmus, S. Tekin, M.B. Aydin, H. Ucar, Ö. Uzun

**Affiliations:** 1 Kirikkale Yuksek Ihtisas Hospital, Psychiatry, Kırıkkale, Turkey; 2 Gulhane Research and Training Hospital, Psychiatry, Ankara, Turkey

**Keywords:** Generalized anxiety disorder, metacognition, obsessive compulsive disorder

## Abstract

**Introduction:**

Generalized anxiety disorder (GAD) and Obsessive compulsive disorder (OCD) are common psychiatric disorders. Researchers studying the pathophysiology of these two disorders evaluated the effect of metacognition. However, there is no research examining the metacognition differences of these two psychiatric conditions.

**Objectives:**

This study was performed to compare the metacognitions in OCD, GAD and healthy controls.

**Methods:**

The sample of this study consisted of 158 GAD and 137 OCD patients aged 18-65 years who presented to outpatient psychiatry clinic and applied to the health committee 168 healthy controls without psychopathology. Sociodemographic data form, Meta-Cognitions Questionnaire-30 scale(MCQ-30), Beck Depression Inventory(BDI) and Beck Anxiety Inventory(BAI) were applied to the volunteer participants who met the criteria for participation in the study. The data obtained were evaluated statistically and subjected to statistical analysis.

**Results:**

The mean age was 31.89 ± 10.86 years and was 60.5% (n = 208) women. There was statistical difference between marital status, occupation and income(p <0.05). In addition, there was a statistically significant difference between MCQ-30 total and subscales, BDI and BAI (p <0.001). According to the comparison of OCD and GAD patients, ’positive belief’, MCQ-30 total and BAI scores were found to be statistically different (p <0.05), ’Uncontrollability and danger’, ’Cognitive Confidence’, ’Beliefs about The Need to Control Thoughts’, ’Cognitive Self-Consciousness’, BDI there was no statistical difference between them (p> 0.05).

**Figure:**
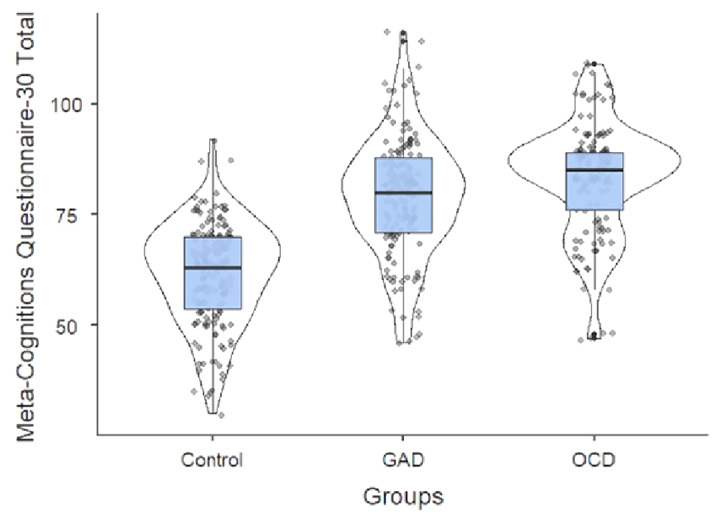

**Conclusions:**

Our results are contributing to the understanding of the uncertainty of development and maintenance of OCD and GAD. Additionally, metacognitions could be important for the diagnosis and treatment of OCD and GAD.

**Disclosure:**

No significant relationships.

